# Profiling of plasma exosome cytokines as biomarkers of severe dengue

**DOI:** 10.3389/fimmu.2026.1779100

**Published:** 2026-04-01

**Authors:** Chien-Tai Hong, Li-Teh Liu, Po-Chih Chen, Chun-Hong Chen, Ching-Yi Tsai, Ping-Chang Lin, Miao-Chen Hsu, Jih-Jin Tsai

**Affiliations:** 1 Department of Neurology, Taipei Medical University-Shuang Ho Hospital, New Taipei, Taiwan; 2 Department of Neurology, School of Medicine, College of Medicine, Taipei Medical University, Taipei, Taiwan; 3 Department of Medical Laboratory Science and Biotechnology, College of Medical Technology, Chung Hwa University of Medical Technology, Tainan, Taiwan; 4 Department of Laboratory Medicine, Kaohsiung Medical University Hospital, Kaohsiung, Taiwan; 5 Department of Medical Laboratory Science and Biotechnology, Kaohsiung Medical University, Kaohsiung, Taiwan; 6 National Mosquito-Borne Diseases Control Research Center, National Health Research Institutes, Miaoli City, Taiwan; 7 National Institute of Infectious Diseases and Vaccinology, National Health Research Institutes, Miaoli City, Taiwan; 8 Tropical Medicine Center, Kaohsiung Medical University Hospital, Kaohsiung, Taiwan; 9 School of Medicine, College of Medicine, Kaohsiung Medical University, Kaohsiung, Taiwan; 10 Division of Infectious Diseases, Department of Internal Medicine, Kaohsiung Medical University Hospital, Kaohsiung, Taiwan

**Keywords:** biomarker, cytokine, dengue fever, exosome, severe dengue

## Abstract

**Introduction:**

Dengue is a global health threat, with severe cases causing significant complications. Cytokines have been proposed as potential indicators of disease severity; however, the short plasma half-life and pre-analytical instability of free-form cytokines limit their clinical applicability. Exosome-encapsulated cytokines are protected by a lipid bilayer and may provide a more stable and integrated representation of host immune responses.

**Methods:**

In this cross-sectional study, we analyzed single time-point plasma samples collected from patients during clinical evaluation between July and December 2023, with most samples obtained during the acute phase (within 7 days post-symptom onset). Plasma exosomes were isolated from patients with mild dengue, dengue with warning signs (DFWS), severe dengue (SD), other febrile illnesses, and healthy controls (HCs). Exosome-associated cytokines were quantified using a multiplex panel assessing 15 cytokines.

**Results:**

Across the five study groups, significant differences in plasma exosome cytokine levels were observed for interleukin (IL)-1β, IL-6, IL-10, IL-12, interferon-γ (IFN-γ), and tumor necrosis factor (TNF)-α (all p < 0.05). Post-hoc analyses further demonstrated that IL-6, IL-10, and TNF-α levels were significantly higher in both the DFWS and SD groups compared with HCs (all p < 0.01). In a subsequent analysis comparing mild dengue with the combined DFWS/SD group, significantly higher exosomal levels of IL-1β, IL-5, IL-10, IL-12, IL-13, and TNF-α were observed in the DFWS/SD group (all p < 0.05). Receiver operating characteristic (ROC) analysis showed that IL-1β, IL-10, and TNF-α moderately discriminated mild from severe cases, with area under the curve (AUC) values of approximately 0.7. An “all-positive” panel (TNF-α, IL-10, and IL-1β) achieved 84% sensitivity and 67.5% specificity for identifying DFWS/SD.

**Conclusion:**

These findings suggest that combined exosomal cytokine profiling may aid in disease severity stratification. However, given the cross-sectional design and moderate discriminatory performance, larger prospective studies are needed to validate its clinical applicability.

## Introduction

Dengue is a major mosquito-borne viral disease and a significant global public health concern, affecting millions of individuals annually, particularly in tropical and subtropical regions (1, 2). While most dengue infections are self-limited, a subset of patients progress to dengue with warning signs (DFWS) or severe dengue (SD), which are associated with plasma leakage, hemorrhage, organ involvement, and increased mortality (3, 4). Early identification of patients at risk of severe disease remains a critical unmet clinical need.

Inflammation plays a crucial role in the pathogenesis and the management of dengue (5–7), and cytokine biomarkers, such as tumor necrosis factor (TNF)-α, and interleukin (IL)-6, have been widely investigated as biomarkers of disease severity (8, 9). However, the clinical utility of free-form plasma cytokines is limited by their short plasma half-life and susceptibility to pre-analytical variability, including degradation during sample handling and storage (10, 11). The instability of cytokines complicates their clinical application as biomarkers, especially in remote or under-resourced regions.

Exosomes are membrane-bound nanoparticles released by cells into bodily fluids. Exosomes carry bioactive molecules such as proteins, lipids, and nucleic acids, reflecting their cellular origin. Exosomes are highly stable in blood due to their lipid membrane, which protects their cargo from enzymatic degradation and changes in the extracellular matrix; thus, exosomes are ideal carriers for molecules serving as biomarkers of infectious diseases (12). In contrast to free-form cytokines, exosome-associated cytokines are encapsulated and are thus less affected by sample handling and storage conditions (13). Although the *in vivo* circulation half-life of endogenous exosomes in humans has not been definitively established, available evidence suggests that exosome-associated molecules may represent a more time-integrated immune signal than free cytokines. Importantly, the apparent disappearance of exosomes from circulation is influenced by biodistribution and cellular uptake rather than direct molecular degradation, and therefore should not be equated with loss of biological stability (13).

Studies have demonstrated that exosome-associated cytokines, such as IL-6, TNF-α, and interferon-gamma (IFN-γ) are associated with the severity of infections including HIV (14), sepsis (15), and COVID-19 (16). Rather than reflecting momentary plasma cytokine concentrations, exosome cargo may capture cumulative immune activation over a clinically relevant time window. This feature is particularly relevant in dengue, where immune responses evolve rapidly during the acute phase of infection.

In the present study, we hypothesized that plasma exosome cytokines serve as novel biomarkers for determining the severity of dengue. Rather than using conventional approaches to investigate a limited number of cytokines, this study utilized a multiplex panel for assessing 15 cytokines simultaneously. This approach enabled the development of a blood exosome cytokine panel with optimal diagnostic accuracy for DFWS and SD. By adopting a cross-sectional design and standardized pre-analytical processing, we sought to explore whether exosome-associated cytokine patterns differ according to dengue severity and whether these profiles show potential utility for severity stratification, rather than establishing causality or clinical prediction.

## Materials and methods

### Sample collection and ethics

This was a cross-sectional study, and the enrollment was conducted between July and December 2023. Participants were recruited from Kaohsiung Medical University Hospital (KMUH), a major referral hospital for dengue cases in Kaohsiung City, Taiwan. In addition, blood sample collection was conducted at KMUH, enabling the collection of comprehensive data and clinical characteristics during dengue outbreaks. Patients with dengue-like symptoms, defined as fever combined with at least two symptoms of headache, retro-orbital pain, muscle or joint pain, rash, nausea, vomiting, or mild hemorrhagic manifestations, were invited to participate in this study. For each participant, a single blood sample was obtained during routine clinical evaluation. The day of symptom onset was recorded, and most samples were collected during the acute phase (within 7 days post symptom onset [PSO]). One sample was obtained on Day 28 from a patient with prolonged symptoms (persistent fever). No longitudinal or repeated sampling was performed. Their plasma samples were collected using plasma separation tubes (Becton Dickinson, USA). Before sample collection, we obtained written informed consent from all participants. For participants who were minors, written informed consent was obtained from their parents or legal guardians. This study was approved by the Institutional Review Board of Kaohsiung Medical University Hospital (Approval No. KMUHIRB-960195).

### Study participants and clinical information

During the 2023 dengue outbreak in Kaohsiung City, plasma samples were collected from 95 participants, comprising those with dengue (n = 65), those with other febrile illnesses (OFI) (n = 15), and healthy controls (HC) (n = 15). Their demographic and clinical data, including age, sex, medical history, symptoms, and laboratory results, were collected. DENV infection was confirmed based on a positive result from real-time polymerase chain reaction (PCR). By contrast, individuals with negative results from PCR and anti-dengue antibody (IgM and IgG) were classified as having OFI. According to the 2009 World Health Organization guidelines, patients were classified into three groups: mild dengue, DFWS (warning signs include abdominal pain, persistent vomiting, fluid accumulation, mucosal bleeding, lethargy, liver enlargement, and an increasing hematocrit with decreasing platelets), and SD (plasma leakage, severe bleeding, or multiorgan involvement) groups.

### Serological classification of primary and secondary infection

Anti-dengue IgG antibodies were measured using the InBios DENV Detect™ IgG ELISA kit according to the manufacturer’s instructions. Following established criteria (17), patients with positive IgG results during the acute phase (≤7 days post symptom onset) were classified as having secondary dengue infection, while IgG-negative patients were classified as primary infection. Samples collected beyond 7 days or yielding indeterminate results were excluded from serostatus-based analyses.

### Sample collection and plasma processing

Peripheral blood samples (10ml from each participant) were collected by venipuncture into EDTA-containing tubes. All samples were processed promptly, and plasma was separated within 3 hours of blood collection by centrifugation at 3,000 × g for 15 minutes to remove cellular components. Plasma aliquots were immediately stored at −80 °C until further analysis. All plasma samples underwent a single freeze–thaw cycle prior to exosome isolation, minimizing potential degradation and pre-analytical variability.

### Exosomes isolation and validation

Exosome isolation was performed after completion of sample collection, and all samples were processed in the same experimental batch to minimize inter-batch variability. Exosomes were isolated from 300 µL storage plasma using the ExoQuick Exosome Isolation and RNA Purification Kit (System Biosciences, Cat No. EXOQ5A-1) following the manufacturer’s protocol. Initially, plasma were centrifuged at 3,000 × g for 15 minutes to remove cellular debris. Plasma samples were pretreated with Thrombin Plasma Prep (System Biosciences, Cat# TMEXO-1) to dissolve fibrin and prevent pellet formation during exosome precipitation. The supernatant was transferred to a sterile tube, and ExoQuick Exosome Precipitation Solution was added at a ratio of 63 µL per 250 µL of sample. For exosome precipitation, the mixture was incubated at 4 °C for 30 minutes. After incubation, the mixture was centrifuged at 1,500 × g for 30 minutes, and the supernatant was discarded. Subsequently, the exosome pellet was resuspended in 60 µL of the provided resuspension buffer for downstream applications.

The isolated exosomes were validated in accordance with the International Society for Extracellular Vesicles 2018 guidelines (18). For this purpose, we performed several characterization assays. Nanoparticle tracking analysis was conducted to quantify the concentration and size distribution of exosomes, thus ensuring consistency in these aspects across samples. Additionally, Western blot analysis was performed to detect exosome-specific markers such as CD63, CD81, and tumor susceptibility gene 101. Transmission electron microscopy analysis confirmed the characteristic morphology of the isolated plasma-derived exosomes, revealing a typical cup-shaped or round vesicular structure with a size range consistent with exosomes (30–150 nm) and the presence of a surrounding lipid bilayer membrane (**Supplementary Figure 1**). These assays confirmed the successful isolation of exosomes that were suitable for subsequent analyses.

### Multiplex panel of inflammatory cytokines

To analyze cytokine levels within exosomes, we employed the Multiplex Human Cytokine HS Screen (15-Plex), a fully quantitative ELISA-based chemiluminescent assay (Boster Biological Technology, MEK1007) following the manufacturer’s instructions. This assay provides the simultaneous quantification of 15 cytokines, as follows: IFN-γ, IL-1α, IL-1β, IL-2, IL-4, IL-5, IL-6, IL-10, IL-12, IL-13, IL-15, IL-17, IL-23, TNF-α, and TNF-β. Briefly, exosome samples were prepared and diluted appropriately. Subsequently, 50 µL of each diluted sample was added to the designated wells in a precoated multiplex plate. The plate was incubated for three hours at room temperature, with gentle shaking to facilitate the binding of cytokines to their respective capture antibodies. After incubation, the wells were washed three times with the provided wash buffer to remove unbound substances. Subsequently, 50 µL of the detection antibody cocktail was added to each well, and the plate was then incubated for one and a half hours at room temperature with shaking. Following washing with the buffer, 50 µL of streptavidin-HRP was added to each well, and the plate was incubated for 15 minutes at room temperature with shaking. After the final washing, 50 µL of the chemiluminescent substrate was added to each well, and the plate was immediately read using Q-View Imager LS (Quansys Biosciences). To determine cytokine concentrations within exosomes, the image data were analyzed using Q-View software (Quansys Biosciences). Each 96-well plate included duplicate measurements for study samples and a complete set of calibration standards to generate standard curves. All samples were assayed under identical conditions.

Cytokine measurements were normalized to an equal input volume of plasma (300 µL per sample), rather than to total exosomal protein content or exosome particle number. This approach was intentionally chosen to reflect the native circulating exosome-associated cytokine burden in patient plasma. Additional normalization based on protein quantity or particle counts was not performed.

### Statistical analyses

Statistical analyses were conducted using SPSS software (version 25.0, IBM Corp., Armonk, NY, USA). Continuous data are expressed as median and ranges. Comparisons of multiple groups were performed using Kruskal–Wallis tests with Dunn’s *post-hoc* analysis. The significance difference between categorical variables was determined using the chi-square test. Multivariable logistic regression was used to obtain odds ratios (ORs) and 95% confidence intervals (CIs) in the multivariate analysis. Receiver operating characteristic (ROC) curve analysis was employed for evaluating the discrimination capability of cytokines and Ct (cycle threshold) value of PCR for dengue severity, specifically for differentiating mild dengue from SD and DFWS. Sensitivity and specificity, positive predictive value, negative predictive value, and overall accuracy were calculated based on the optimal cutoff values determined from the ROC analysis. A p-value of <0.05 was considered statistically significant.

## Results

### Characteristics of dengue, OFI, and HC groups

The demographic characteristics of the study participants are summarized in **Table 1**. Significant differences in age distribution were observed among patients with dengue, individuals with other febrile illnesses (OFI), and healthy controls (HC) (p = 0.032). When stratified into three age categories (<20 years, 20–59 years, and ≥60 years), patients with dengue showed a higher proportion of older adults, with 31 of 65 patients (47.7%) aged ≥60 years, compared with 6 of 15 (40.0%) in the OFI group and 2 of 15 (13.3%) in the HC group. In contrast, HC participants were predominantly within the 20–59-year age group. The distribution across these three age categories differed significantly between groups (p = 0.011). Female sex was more common among patients with dengue than in the OFI group (56.9% vs. 20.0%, p = 0.018). No significant difference was observed in the number of days post symptom onset (PSO) between the dengue and OFI groups.

**Table 1:** Demographic information of study participants.

Variables	Dengue, n=65	Other febrile illness, n=15	Healthy controls, n=15	P value
Age, median (range)	54 (7-88)	57 (3-82)	34 (20-63)	0.032
<20 years old	3	3	0	
20–59 years old	31	6	13	0.011
≥ 60 years old	31	6	2	
Female %	37 (56.9)	3 (20)	10 (66.7)	0.018
Days PSO, median (range)	1 (0-28)	3 (0-11)	N/A	0.113
Infection status				
Primary infection	38	N/A	N/A	N/A
Secondary infection	24	N/A	N/A	
Indeterminate	3	N/A	N/A	
Serotype of DENV				
Type 1	30	N/A	N/A	N/A
Type 2	35	N/A	N/A	

DENV, dengue virus; PSO, post symptom onset; N/A, Not applicable; healthy controls and other febrile illness did not have disease onset, infection status or identifiable pathogens. Indeterminate status: Refers to inconclusive ELISA results and samples collected >7 days post-onset.

### Differential exosome cytokine levels among study groups

The plasma exosome cytokine levels in the five study groups—HC, OFI, mild dengue, DFWS, and SD—are summarized as the median in **Table 2**. The severe dengue group was relatively small (n = 8), reflecting the epidemiological distribution during the study period. Significant differences for the trend were observed across the groups in multiple cytokines, including IL-1β, IL-6, IL-10, IL-12, IFN-γ, and TNF-α. Based on Dunn’s *post-hoc* analysis, plasma exosome IL-6 levels were significantly lower in the HC group compared with mild dengue (p < 0.001), OFI (p = 0.010), DFWS (p < 0.001), and SD (p = 0.003). Similarly, IL-10 levels were significantly lower in HC compared with mild dengue (p < 0.001), OFI (p = 0.010), DFWS (p < 0.001), and SD (p = 0.003). Plasma exosome IL-12 levels were significantly lower in HC compared with mild dengue (p = 0.004) and DFWS (p < 0.001). In addition, TNF-α levels were significantly lower in HC compared with DFWS (p = 0.001) and SD (p = 0.003). Plasma exosome IFN-γ levels were significantly lower in HC compared with mild dengue (p < 0.001), OFI (p = 0.010), DFWS (p < 0.001), and SD (p = 0.003). However, not all proinflammatory cytokines demonstrated uniform increases across severity categories. For example, IFN-γ levels were lower in the SD group compared with DFWS, and several cytokines (e.g., IL-12, IL-17, and IL-1α) did not differ significantly from controls. In similar, not all cytokines included in the multiplex panel demonstrated statistically significant differences across severity categories, indicating heterogeneity in exosome-associated cytokine responses. Among patients with available serotype information, 30 were infected with DENV-1 and 35 with DENV-2. No significant differences were observed in exosome-associated cytokine levels between DENV-1 and DENV-2 infections for any of the 15 cytokines analyzed (all p > 0.05, **Supplementary Table 1**).

**Table 2:** Plasma exosome cytokine levels (median) in different groups of study participants.

	HC	OFI	Mild dengue	SD	DFWS		
pg/ml (plasma)	n=15	n=15	n=40	n=8	n=17	P-value for trend	*Post-hoc* analysis
IL-1α	19.25	20.6	18.675	19.375	21.4	0.681	
IL-1β	38.8	67	38.825	67.875	38.2	0.029	
IL-2	0.9	1.5	1.225	2.4	2.05	0.079	
IL-4	0.22	0.225	0.2175	0.225	0.225	0.744	
IL-5	5.1	5.9	4.6	6.75	7.1	0.146	
IL-6	3.25	15.7	14.65	103.85	18.1	<0.001	*a*
IL-10	28.6	35.95	38.775	103.925	89.5	<0.001	*a*
IL-12	31	32.8	34.575	38.175	38.95	<0.001	*b*
IL-13	5.75	4.7	4.825	6.4	5.1	0.06	
IL-15	50.6	49.7	49.4	55.15	50.95	0.724	
IL-17	67.4	62.6	65.55	73.65	64.1	0.393	
IL-23	251.2	286.5	277.1	304.25	303.55	0.298	
IFN-γ	1.3	6.85	13.2	4.2	14.9	<0.001	*c*
TNF-α	9.7	16.9	16.2	45.6	24.05	<0.001	*b*
TNF-β	6.0	6.15	5.4	5.65	4.7	0.587	

DFWS, Dengue with warning signs; HC, Healthy control; IFN, interferon; IL, interleukin; OFI, Other febrile illness; SD, Severe Dengue; TNF, tumor necrosis factor. For *post-hoc* analysis, *a*, significant difference between HC with all other groups; *b*, significant difference between HC with mild dengue, SD and DFWS; *c*, significant difference between HC with DFWS, mild dengue and OFI.

### Exosomal cytokine differences between mild dengue and combined DFWS/SD groups

**Figure 1:**
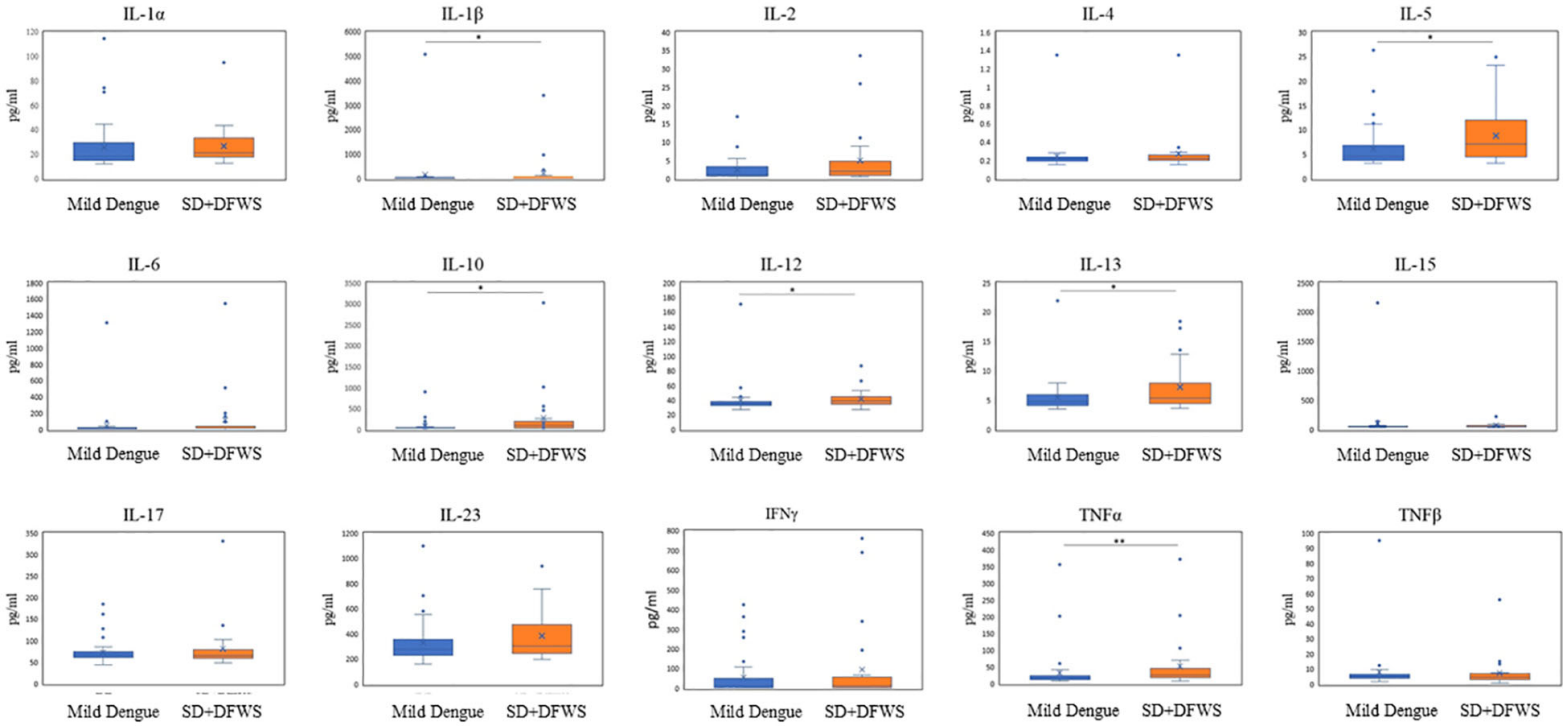
The comparison of plasma exosome cytokines between two groups of study participants with dengue. (DF, Dengue fever; DFWS, Dengue with warning signs; IFN, interferon; IL, interleukin; SD, Severe Dengue; TNF, tumor necrosis factor). *p<0.05; **p<0.01.

Further comparisons of the levels of plasma exosome cytokines were conducted between the mild dengue group (n = 40) and the combination of SD and DFWS groups (n = 25). The cytokine levels in the SD and DFWS groups were significantly higher than those in the mild dengue group. Significant differences were observed in the levels of plasma exosome IL-1β, IL-5, IL-10, IL-12, IL-13, and TNF-α between the mild dengue group and the SD and DFWS groups. The most significant differences were found in TNF-α levels (p=0.005). No significant differences were observed in the levels of the other cytokines between the mild dengue group and the SD and DFWS groups (**Figure 1**).

### Analysis of clinical characteristics and Ct value as predictors of dengue severity

To assess whether demographic and clinical factors might confound differences in plasma exosome cytokines, we compared patients with mild dengue to the combined group with SD or DFWS. Baseline demographic and clinical characteristics of patients with mild dengue and those with DFWS/SD are summarized in **Table 3**. No significant differences were observed between the two groups in age (p = 0.905), sex (p = 0.362), dengue serotype (p = 0.431), infection status (p = 0.360), comorbidities, or most laboratory parameters, including hematocrit (p = 0.442) and platelet count (p = 0.074). Alanine aminotransferase (ALT; also known as glutamate–pyruvate transaminase, GPT) levels were significantly higher in the DFWS/SD group (median 59 U/L vs. 31 U/L, p = 0.038). To identify independent factors associated with DFWS/SD, a multivariable logistic regression analysis was performed (**Table 4**). After adjustment, ALT was no longer significantly associated with disease severity (adjusted OR = 1.005, 95% CI: 0.995–1.014, p = 0.335), whereas a PCR Ct value ≥20.54 remained independently associated with DFWS/SD (adjusted OR = 4.41, 95% CI: 1.52–12.86, p = 0.005).

**Table 3:** Demographic and clinical factors between mild dengue with the combination of dengue with warning signs (DFWS) and severe dengue (SD).

Demographic characteristics	Mild dengue,	DFWS+SD,	P-value
	n=40	n=25	
Age ≥ 65 years old	17 (42.5)	11 (44)	0.905
Female	21 (52.5)	16 (64)	0.362
Serotype			
DENV-1	20 (50)	10 (40)	0.431
DENV-2	20 (50)	15 (60)	
Infection status ^a^			
Primary	25 (65.8)	13 (54.2)	0.360
Secondary	13 (34.2)	11 (45.8)	
Ct of PCR	17.96 (12.75-32.3)	21.87 (15.31-37.88)	0.007
≥20.54	13(32.5)	17(68)	0.005
CRP	7.39 (0.79-75.14)	9.36 (2.33-163.41)	0.196
Days PSO	1 (0-11)	2(0-28)	0.124
Chronic disease			
CCI	0 (0-5)	1 (0-5)	0.265
Diabetes mellitus	7 (17.5)	9 (36)	0.092
Hypertension	15 (37.5)	10 (40)	0.840
CHF	1 (2.5)	1 (4)	0.733
COPD	1 (2.5)	0	0.426
CVD	2 (5)	1 (4)	0.852
CKD stage 4~5	3 (7.5)	3 (12)	0.542
Malignancy	2 (5)	3 (12)	0.303
Hyperlipidemia	0	1 (4)	0.202
Length of stay (days)	6 (0-13)	5 (0-34)	0.429
Laboratory Characteristics			
Hematocrit (%)	39.45 (28.2-46.3)	38.1 (20.7-49.5)	0.442
Platelet count (x1000/uL)	137.5 (22-401)	91 (8-401)	0.074
WBC (x1000/uL)	3.42 (1.4-16.42)	4.13 (0.53-18.13)	0.230
ALT (IU/L)	31 (8-273)	59 (10-801)	0.038
Creatinine (mg/dL)	0.81 (0.58-1.43)	0.77 (0.55-2.79)	0.600

ALT, Alanine Aminotransferase; CCI, Charlson comorbidity index score; CHF, congestive heart failure; CKD, chronic kidney disease; COPD, chronic pulmonary disease; Ct of PCR, threshold cycles of polymerase chain reaction, CRP, C-reactive protein; CVD, cerebrovascular disease; DENV, dengue virus; PSO, post symptom onset. WBC, White blood cell. Categorical variables are expressed as number (%), and continuous variables are expressed as median (range).

**Table 4:** Multivariable logistic regression analysis for factors associated with the combination of dengue with warning signs (DFWS) and severe dengue (SD).

Variable	Adjusted OR	95% CI	P value
Age ≥65 years	1.06	0.39–2.91	0.905
Female sex	1.61	0.58–4.49	0.362
DENV-2 (vs DENV-1)	1.50	0.55–4.13	0.431
Secondary infection	0.62	0.22–1.74	0.360
PCR Ct ≥20.54	4.41	1.52–12.86	0.005
ALT (per 1 U/L increase)	1.005	0.995–1.014	0.335
Platelet count	0.997	0.987–1.007	0.074
Hematocrit	0.982	0.882–1.094	0.442

ALT, Alanine Aminotransferase; Ct of PCR, threshold cycles of polymerase chain reaction, DENV, dengue virus; PSO, post symptom onset.

### ROC analysis and diagnostic panel development using exosomal cytokines

**Figure 2:**
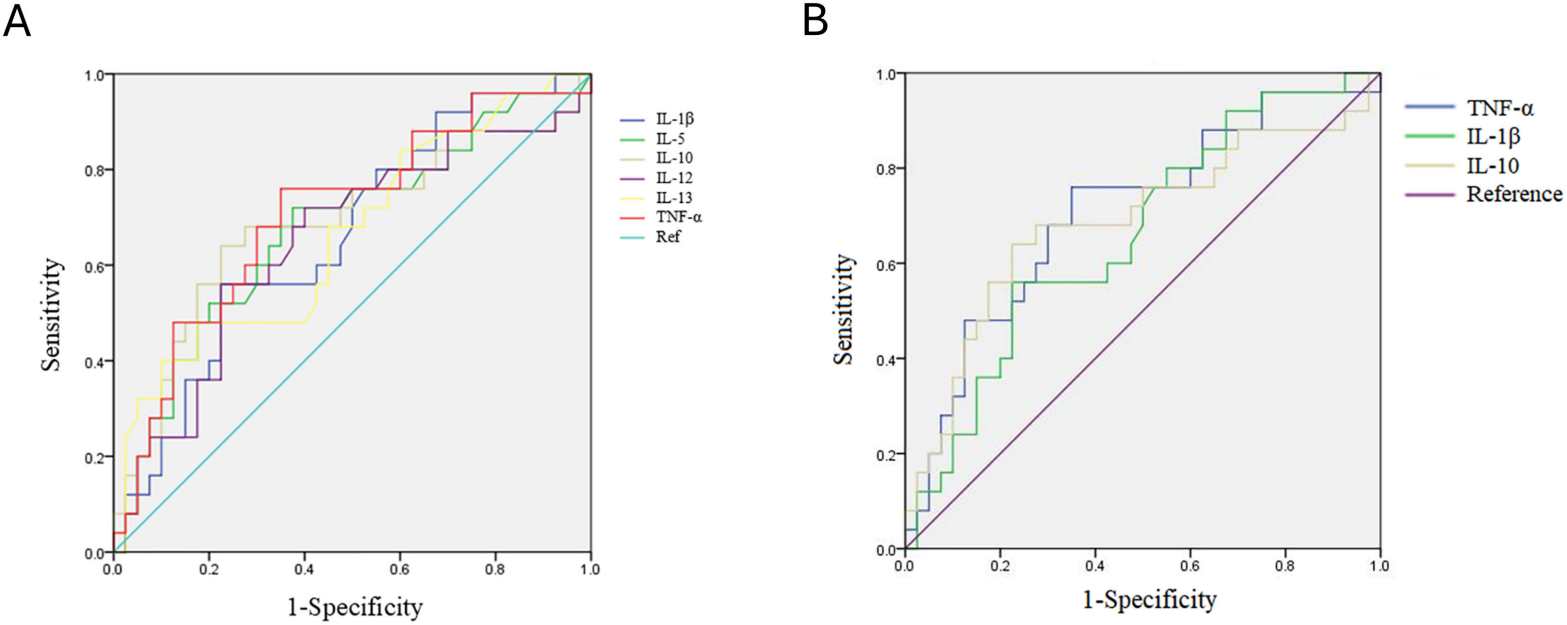
Receiver operating characteristic (ROC) curve analysis of plasma exosome cytokines for distinguishing mild dengue from more severe forms (dengue with warning signs and severe dengue). **(A)** Cytokines that significantly differentiate mild from severe dengue. **(B)** The top three cytokines with the highest area under the curve (AUC) values.IL, interleukin; TNF, tumor necrosis factor.

ROC curve analyses were conducted to evaluate the capability of these cytokines for discriminating mild dengue from DFWS and SD from the six cytokines with significantly difference between mild dengue and the SD+DFWS group (**Figure 2A**). Plasma exosome IL-1β, IL-10, and TNF-α levels achieved significant discrimination capability, with ROC area under the curve (AUC) values ranging from 0.66 to 0.707 (**Figure 2B**). These analyses were exploratory in nature and were not intended to establish diagnostic thresholds.

Based on the results, combination panels for dengue diagnosis were tested. Firstly, the “any-positive” diagnostic panel, which considers cases positive when any one of the three most associated cytokines exceeds a high threshold (TNF-α ≥ 13.75pg/ml, IL-10 ≥ 35.075pg/ml, IL-1β ≥ 27.4pg/ml), which offered a decent sensitivity (88%), ensuring the detection of nearly all severe cases, but alongside a compromised specificity (30-37.5%). On the other hand, another panel required all positive results, in which the levels of all three cytokines exceeded moderate cutoff values simultaneously (TNF-α ≥ 13.75 pg/mL plasma, IL-10 ≥ 35.425 pg/mL plasma, IL-1β ≥ 25.80 pg/mL plasma). These cut-off values for each exosomal cytokine solely provided high sensitivity but low specificity for distinguishing DFWS+SW between mild dengue (**Supplementary Table 2**). However, in combination, the “all-positive” panel preserved the satisfactory sensitivity (84%) and reached a superior specificity (67.5%) (**Table 5**). This combined approach improved sensitivity compared with individual cytokines, although specificity remained moderate. These findings indicate potential utility for severity stratification but do not establish clinical diagnostic performance.

**Table 5:** Diagnostic sensitivity and specificity of moderate cut-off value for plasma exosome cytokines.

	TNF-α	IL-10	IL-1β	All positive (n.)	
Cut-off (pg/ml plasma)	≥13.75	≥35.425	≥25.80		
Mild Dengue (n=40)	25	27	31	13	Specificity 67.5% (27/40)
DFWS+SD (n=25)	22	21	24	21	Sensitivity 84% (21/25)

DFWS, Dengue with warning signs; IL, interleukin; SD, Severe Dengue; TNF, tumor necrosis factor.

Further analysis revealed that there was no significant difference noted in demographic characteristics, comorbidity, and blood CRP levels between the true positive and false negative groups of DFWS and SD patients and between the false positive and true negative groups of mild dengue patients (**Supplementary Table 3**, **S4**).

## Discussion

The present study demonstrates that plasma exosome–associated cytokines show distinct distribution patterns across dengue severity categories. Using a standardized, single time-point sampling strategy, we observed higher levels of several exosome-encapsulated cytokines—particularly TNF-α, IL-10, and IL-1β—in patients with DFWS and SD compared with mild dengue. These findings suggest that exosome-associated cytokine profiling may provide complementary information to conventional clinical and laboratory markers for severity stratification. Rather than establishing a definitive diagnostic tool, the current results support the potential utility of exosome-associated cytokines as severity-associated biomarkers in dengue. The moderate discriminatory performance observed in ROC analyses (AUC approximately 0.7) indicates that these markers are hypothesis-generating and warrant further validation in larger and longitudinal cohorts.

Inflammatory cytokines such as TNF-α and IL-10 play well-established roles in dengue pathogenesis, particularly in endothelial dysfunction, vascular leakage, and immune dysregulation (19–21). However, the clinical interpretation of free-form plasma cytokines is complicated by their short circulating half-lives and substantial temporal fluctuations during acute infection (22–24). Exosome-encapsulated cytokines differ fundamentally from free cytokines in circulation. While the *in vivo* half-life of endogenous exosomes in human plasma has not been definitively quantified, existing evidence indicates that the apparent clearance of exosomes from blood is largely driven by biodistribution and cellular uptake, rather than rapid molecular degradation (25, 26). Consequently, the absence of exosomes from circulation does not necessarily imply loss of biological stability. Importantly, the stability of exosomes ex vivo has been well documented. In this study, blood samples were processed rapidly, and plasma was stored at −80 °C prior to batch exosome isolation, conditions under which exosome integrity and cargo stability have been shown to be preserved. Therefore, the measured exosome-associated cytokine profiles are unlikely to be artifacts of pre-analytical degradation.

TNF-α is a central mediator of dengue-associated inflammation and vascular permeability (27, 28). Although free TNF-α has an estimated plasma half-life of only minutes (29), TNF-α encapsulated within exosomes may reflect a more integrated immune signal accumulated over a clinically relevant time window. In the present cohort, plasma exosome TNF-α levels increased progressively with disease severity and showed moderate ability to distinguish mild dengue from DFWS/SD.

Similarly, IL-10 is a key immunoregulatory cytokine implicated in severe dengue (30, 31), but half-life is short as well (32). Elevated exosome-associated IL-10 levels in DFWS and SD patients may reflect sustained anti-inflammatory signaling accompanying dysregulated immune activation. These observations should be interpreted as associations with disease severity, rather than evidence of causality or temporal prediction.

Recent studies in viral infections suggest that multi-marker approaches outperform single biomarkers by capturing the complexity of host immune responses (33, 34). In line with this concept, the combined panel of TNF-α, IL-10, and IL-1β achieved higher sensitivity than individual cytokines alone. However, the moderate specificity and cross-sectional design of this study preclude immediate clinical application. The proposed panel should be viewed as a proof-of-concept tool for severity stratification, rather than a validated diagnostic or prognostic assay. However, the study did not construct or validate a multi-cytokine diagnostic panel.

This study provides a comprehensive characterization of plasma exosome–associated cytokine profiles across dengue severity categories using a multiplex approach. By simultaneously assessing multiple pro- and anti-inflammatory cytokines encapsulated within exosomes, our findings offer additional insight into immune dysregulation during dengue infection. The inclusion of both healthy controls and patients with other febrile illnesses allowed a more specific evaluation of dengue-associated immune signatures. Moreover, comparison across different clinical severity groups enabled exploration of severity-related cytokine patterns. While exploratory in nature, the multi-cytokine panel approach demonstrated moderate discriminatory performance for disease severity stratification, supporting its potential value as a complementary research tool rather than a definitive clinical predictor.

This study has several important limitations. First, its cross-sectional design and single time-point sampling prevent assessment of temporal cytokine dynamics and preclude causal inference. Second, the sample size—particularly in the severe dengue subgroup—limits statistical power and generalizability. Third, age distribution differed across groups, and although sensitivity analyses were performed and reached similar results, residual confounding cannot be fully excluded. Future studies with larger, independent cohorts are needed to validate age-specific biomarker profiles. Furthermore, although certain cytokines differed between severe dengue and mild dengue, substantial overlap was observed between dengue and OFI groups. Because OFI represents systemic inflammatory conditions, differentiation between dengue and other febrile illnesses based solely on exosome-associated cytokines remains challenging. Therefore, the proposed panel is better interpreted as a tool for severity stratification among confirmed dengue cases rather than for primary diagnostic discrimination. Finally, exosome isolation and multiplex cytokine assays currently require laboratory infrastructure that may not be readily available in resource-limited settings where dengue is endemic.

## Conclusion

The present study demonstrated that plasma exosome-associated cytokines demonstrate distinct patterns across dengue severity categories and may reflect integrated immune activation during acute infection. A combined panel of TNF-α, IL-10, and IL-1β showed moderate performance for severity stratification. These findings support further prospective and longitudinal studies to validate exosome-based cytokine profiling as a complementary approach in dengue research.

## Data Availability

The original contributions presented in the study are included in the article/**Supplementary Material**. Further inquiries can be directed to the corresponding author.
