# Supplementary material for: Profiling of plasma exosome cytokines as biomarkers of severe dengue
Source: Front Immunol. 2026 Apr 1;17:1779100. doi: 10.3389/fimmu.2026.1779100 (PMC13079002; doi:10.3389/fimmu.2026.1779100)
Supplement: Supplementary file 1 [file DataSheet1.docx]

Supplementary Information

Supplementary Table S1. Median plasma exosome cytokine levels in patients with DENV-1 versus DENV-2 infection.

| Cytokines | DENV-1 | DENV-2 | p value |
| --- | --- | --- | --- |
| (pg/ml) | n=30 | n=35 |  |
| IL-1α | 19.400 | 20.050 | 0.990 |
| IL-1β | 40.800 | 39.900 | 0.626 |
| IL-2 | 1.475 | 1.300 | 0.995 |
| IL-4 | 0.210 | 0.230 | 0.161 |
| IL-5 | 5.300 | 5.150 | 0.849 |
| IL-6 | 14.450 | 19.950 | 0.554 |
| IL-10 | 44.100 | 45.050 | 0.864 |
| IL-12 | 37.525 | 34.600 | 0.108 |
| IL-13 | 5.000 | 5.300 | 0.984 |
| IL-15 | 48.650 | 51.150 | 0.655 |
| IL-17 | 66.000 | 65.400 | 0.317 |
| IL-23 | 306.050 | 276.450 | 0.757 |
| IFN-γ | 13.700 | 12.650 | 0.418 |
| TNF-α | 21.575 | 17.500 | 0.603 |
| TNF-β | 5.550 | 5.350 | 0.485 |

Supplementary Table S2. Diagnostic sensitivity and specificity for moderate cut-off value for each selected blood exosome cytokine

|  | TNF-α | IL-10 | IL-1β |
| --- | --- | --- | --- |
| Cut-off  (pg/ml plasma) | ≥13.75 | ≥35.425 | ≥25.80 |
| Specificity | 37.5% (15/40) | 32.5% (13/40) | 22.5% (9/40) |
| Sensitivity | 88% (22/25) | 84% (21/25) | 96% (24/25) |

Supplementary Table S3. The comparison between mild dengue patients with/without “All-Positive” of blood exosomal cytokine panel.

| Variables | All Positive, | non-All Positive, | p-value |
| --- | --- | --- | --- |
|  | n=13 | n=27 |  |
| Age | 61(25-88) | 51 (20-87) | 0.977 |
| Female | 7 | 14 | 0.906 |
| PSO (days) | 1 (0-11) | 1 (0-5) | 0.448 |
| CCI | 0 (0-4) | 0 (0-5) | 0.929 |
| CRP | 8.69 (1.14-75.14) | 5.69 (0.79-44.75) | 0.413 |
| Ct of PCR | 21.30 (15.11-30.70) | 17.07 (12.75-32.30) | 0.109 |
| Serotype |  |  |  |
| DENV-1 | 8 | 12 | 0.311 |
| DENV-2 | 5 | 15 |  |
| Primary infection | 7 | 19 | 0.265 |
| Secondary infection | 5 | 8 |  |
| Indeterminate | 1 | 0 |  |

Data were presented as median (range). CCI, Charlson Comorbidity Index; CRP, C-reactive protein; Ct of PCR, threshold cycles of polymerase chain reaction; PSO, post symptom onset

Supplementary Table S4. The comparison between severe dengue patients with/without “All-Positive” of blood exosomal cytokine panel.

| Variables | All Positive, | non-All Positive, | p value |
| --- | --- | --- | --- |
|  | n=21 | n=4 |  |
| Age | 56 (7-86) | 54 (21-73) | 0.853 |
| Female | 14 | 2 | 0.524 |
| PSO days | 2 (0-7) | 1 (0-28) | 0.848 |
| CCI | 1 (0-5) | 1 (0-2) | 0.877 |
| CRP | 13.5 (2.33-163.41) | 8.42 (5.31-9.36) | 0.273 |
| Ct of PCR | 21.51 (15.31-37.88) | 21.88 (21.19-31.37) | 0.553 |
| Serotype |  |  |  |
| DENV-1 | 9 | 1 | 0.504 |
| DENV-2 | 12 | 3 |  |
|  |  |  |  |
| Primary infection | 10 | 3 | 0.315 |
| Secondary infection | 11 | 1 |  |

Data were presented as median (range). CCI, Charlson Comorbidity Index; CRP, C-reactive protein; Ct of PCR, threshold cycles of polymerase chain reaction; PSO, post symptom onset

Supplementary Figure S1. Characterization of isolated serum exosome. (A) Nanoparticle tracking analysis of size distribution of the isolated exosomes from different study participants. (B) Representative western blot images of the differential expression of exosomal markers CD63, CD81, and TSG101 in the isolated serum exosomes from different study participants. HSP70 was served as loading control. (C) Characterization of blood exosome morphologically by transmission electron microscopy.


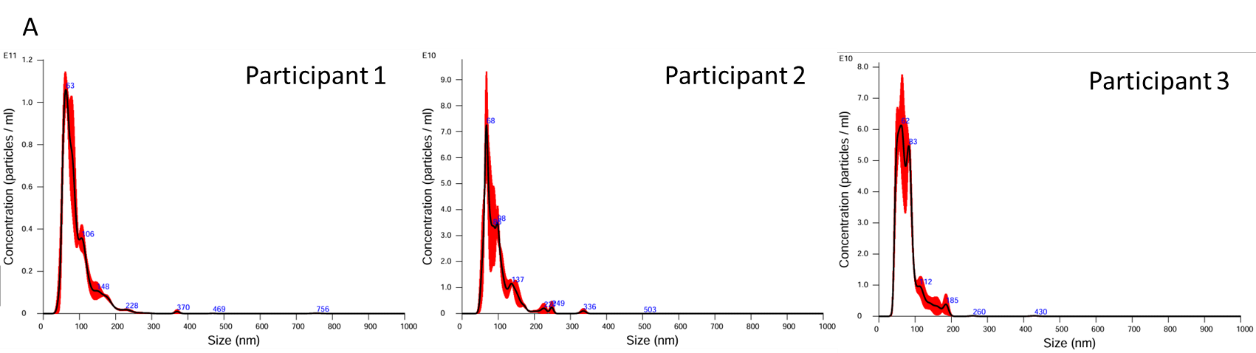


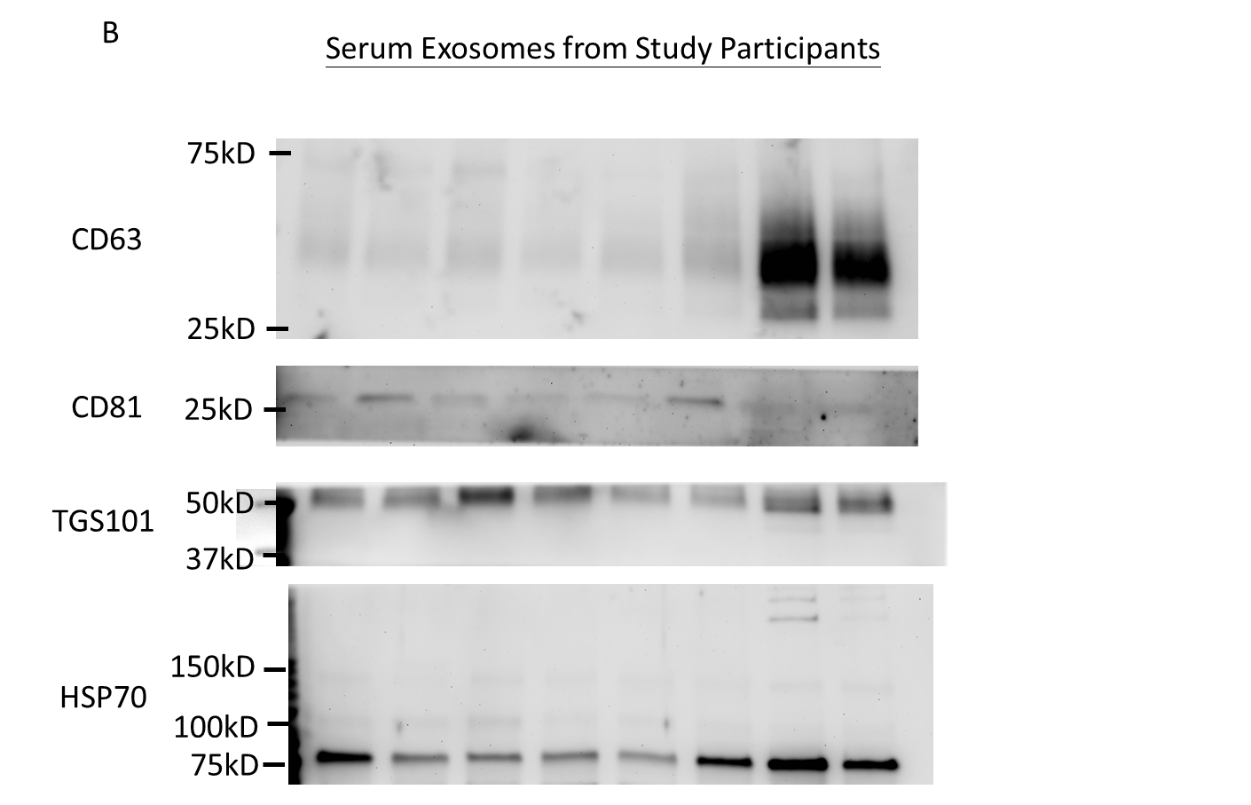


C


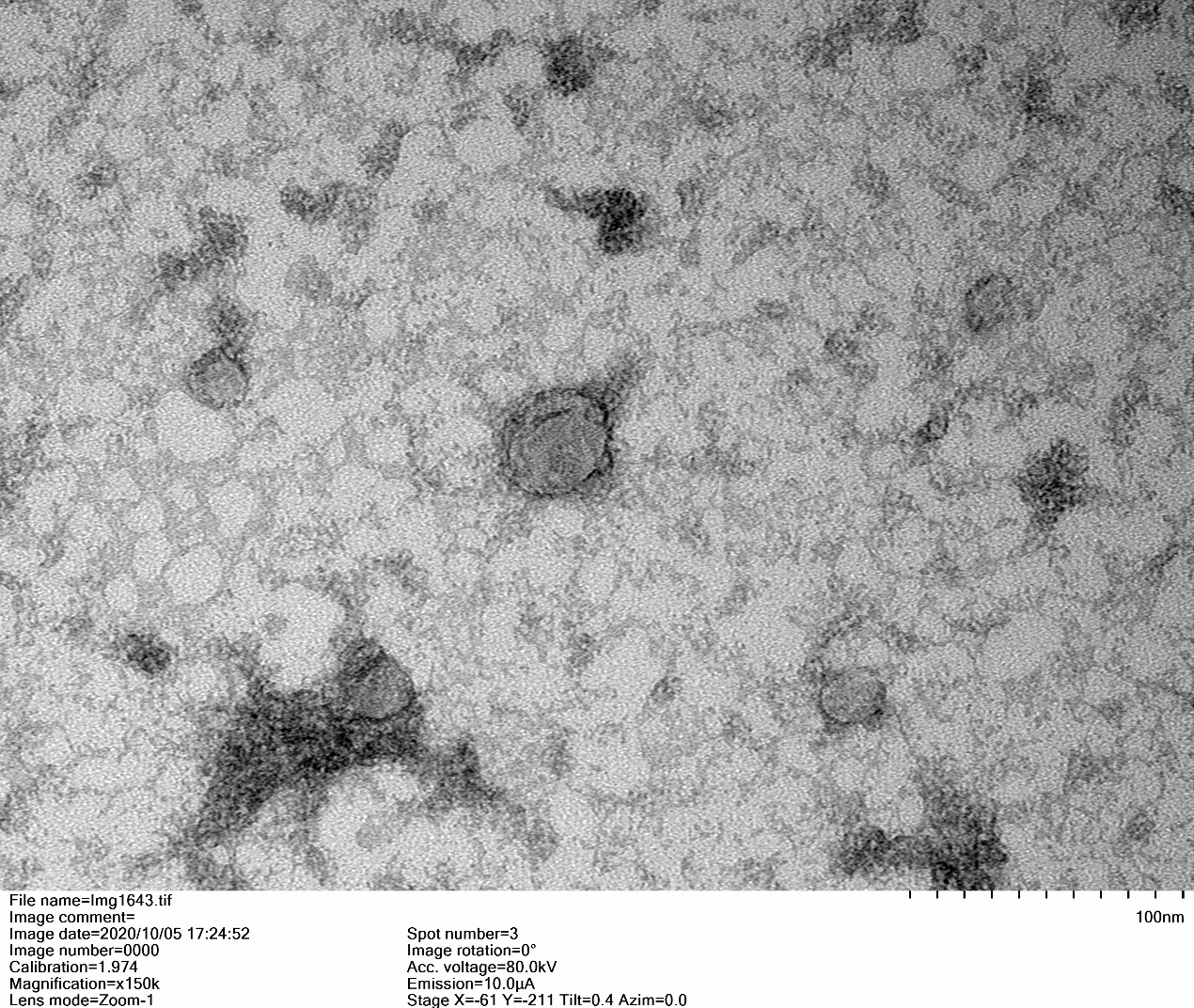

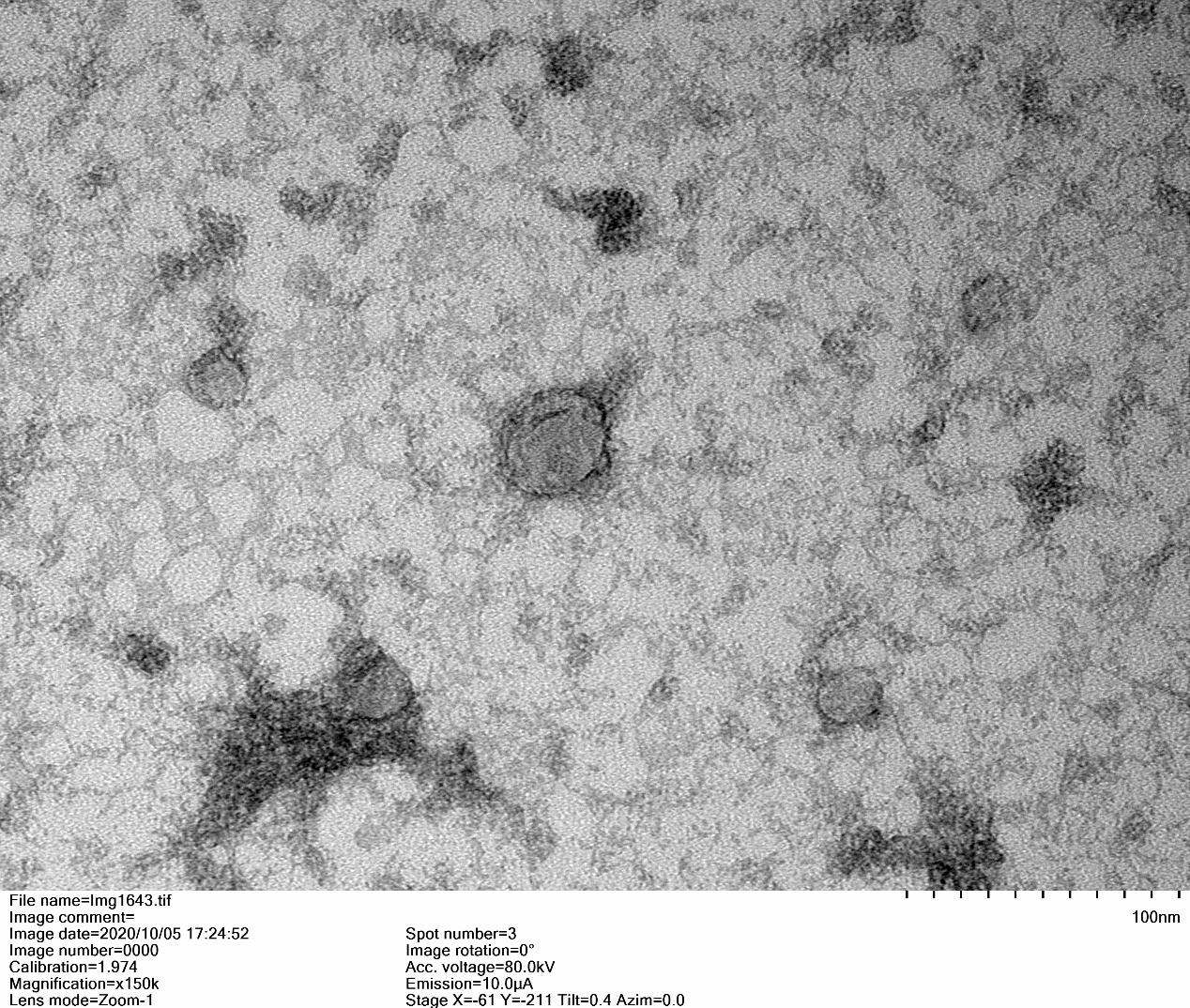


***Methods of Exosome Isolation and Validation***

Exosomes were isolated from 300 µL storage plasma using the ExoQuick Exosome Isolation and RNA Purification Kit (System Biosciences, Cat No. EXOQ5A-1) following the manufacturer's protocol. The detail was provided in the supplementary information. Initially, plasma were centrifuged at 3,000 × g for 15 minutes to remove cellular debris. Plasma samples were pretreated with Thrombin Plasma Prep (System Biosciences, Cat# TMEXO-1) to dissolve fibrin and prevent pellet formation during exosome precipitation. The supernatant was transferred to a sterile tube, and ExoQuick Exosome Precipitation Solution was added at a ratio of 63 µL per 250 µL of sample. For exosome precipitation, the mixture was incubated at 4°C for 30 minutes. After incubation, the mixture was centrifuged at 1,500 × g for 30 minutes, and the supernatant was discarded. Subsequently, the exosome pellet was resuspended in 60 µL of the provided resuspension buffer for downstream applications.

The isolated exosomes were validated in accordance with the International Society for Extracellular Vesicles 2018 guidelines. For this purpose, we performed several characterization assays. Nanoparticle tracking analysis was conducted to quantify the concentration and size distribution of exosomes, thus ensuring consistency in these aspects across samples. Additionally, Western blot analysis was performed to detect exosome-specific markers such as CD63, CD81, and tumor susceptibility gene 101. Transmission electron microscopy analysis confirmed the characteristic morphology of the isolated plasma-derived exosomes, revealing a typical cup-shaped or round vesicular structure with a size range consistent with exosomes (30–150 nm) and the presence of a surrounding lipid bilayer membrane (Supplementary Figure 1). These assays confirmed the successful isolation of exosomes that were suitable for subsequent analyses.

***Multiplex Panel of Exosome Inflammatory Cytokines***

Exosome samples were prepared and diluted appropriately. Subsequently, 50 µL of each diluted sample was added to the designated wells in a precoated multiplex plate. The plate was incubated for three hours at room temperature, with gentle shaking to facilitate the binding of cytokines to their respective capture antibodies. After incubation, the wells were washed three times with the provided wash buffer to remove unbound substances. Subsequently, 50 µL of the detection antibody cocktail was added to each well, and the plate was then incubated for one and a half hours at room temperature with shaking. Following washing with the buffer, 50 µL of streptavidin-HRP was added to each well, and the plate was incubated for 15 minutes at room temperature with shaking. After the final washing, 50 µL of the chemiluminescent substrate was added to each well, and the plate was immediately read using Q-View Imager LS (Quansys Biosciences). To determine cytokine concentrations within exosomes, the image data were analyzed using Q-View software (Quansys Biosciences), and the sample readings were compared to a standard curve generated from known concentrations of each cytokine provided in the kit.
